# Relationship of Hobby Activities With Mortality and Frailty Among Community-Dwelling Elderly Adults: Results of a Follow-up Study in Japan

**DOI:** 10.2188/jea.JE20110057

**Published:** 2012-07-05

**Authors:** Yasuhiro Fushiki, Hirofumi Ohnishi, Fumio Sakauchi, Asae Oura, Mitsuru Mori

**Affiliations:** 1 Department of Public Health, Sapporo Medical University School of Medicine, Sapporo, Japan; 2 Department of Genome Medicine, National Cerebral and Cardiovascular Center, Suita, Osaka, Japan

**Keywords:** elderly people, hobby activities, cultural activities, physical activities, mortality, frailty

## Abstract

**Background:**

The proportion of Japanese adults aged 65 years or older is predicted to increase; thus, it is becoming more important to identify factors that influence health status among elderly adults in Japan. We conducted a follow-up study of community-dwelling elderly adults to assess the relationship of hobby activities with mortality and incident frailty.

**Methods:**

We randomly selected 3583 individuals aged 65 to 84 years from the residential registries of 7 study areas in Hokkaido, Japan in August 2007. Among them, 1955 (54.6%) returned completed questionnaires with written informed consent by mail. The baseline assessment questionnaire comprised questions on current and past involvement in hobby activities, self-perceived health status, smoking and drinking habits, and body height and weight. Questions on hobby activities were from 4 categories: solitary physical, group physical, solitary cultural, and group cultural activities. We later conducted a follow-up survey of the participants to ascertain all-cause mortality and incident frailty. A Cox proportional hazards model was used for analysis of data from September 2007 to May 2010.

**Results:**

After adjusting for potential confounders, the risk of incident frailty among respondents participating in solitary physical activities was significantly lower than in those who did not participate in such activities (hazard ratio = 0.57; 95% CI 0.33, 0.99). Furthermore, the risk of incident frailty among respondents taking part in group cultural activities was significantly lower than in those who did not participate in such activities (0.41; 0.19, 0.87).

**Conclusions:**

These findings may be important for programs that seek to promote good health among elderly adults.

## INTRODUCTION

According to the 2009 Japanese abridged life table, life expectancy at birth was 79.6 years and 86.4 years for males and females, respectively,^1^ which are among the highest life expectancies in the world.^2^ In contrast, the number of births has been decreasing in Japan. As a result, the proportion of people aged 65 years or older is predicted to be 26.9% by 2015 and 40.5% by 2055.^3^ Therefore, it has become more important to identify factors that influence health status among elderly adults in Japan. The Japanese National Government, acting with local governments, has recently developed a social campaign to promote well-being and health in elderly adults. Consequently, 59.2% of persons aged 60 years or older participated in certain group activities in 2008, as compared with 44.3% in 1998. These group activities were classified as physical/sports activities (30.5%), activities for local events (24.4%), and cultural hobby activities (20.2%).^4^

Physical activity is inversely associated with rates of mortality^5^^–^^11^ and impaired health.^12^^,^^13^ Similarly, it has been suggested that regular participation in cultural activities is associated with lower rates of mortality^14^^–^^16^ and impaired health.^17^^,^^18^

We classified physical and cultural activities into 2 categories—solitary and group activities—and tested 2 hypotheses regarding elderly adults: (1) cultural activities and physical activities would similarly decrease the risks of mortality and incident frailty, and (2) solitary activities and group activities would similarly decrease the risks of mortality and incident frailty. To evaluate these hypotheses, we conducted a follow-up study of community-dwelling elderly adults to assess the relationships of hobby activities with mortality and incident frailty. Hobby activities were categorized as solitary physical, group physical, solitary cultural, and group cultural activities.

## METHODS

This follow-up study enrolled community-dwelling elderly adults residing in 7 study areas located in central Hokkaido Island, northern Japan, including Ebetsu City, Iwamizawa City, Kitahiroshima City, Ishikari City, Tobetsu Town, Kuriyama Town, and Naganuma Town. These 7 areas around Sapporo city were chosen because they were easily accessible from our university. This study was approved by the Ethics Committee of Sapporo Medical University.

We randomly selected 3583 study candidates aged 65 to 84 years, after excluding those with addresses corresponding to hospitals or nursing homes. Almost 500 persons per study area were identified on residential registries of these study areas in August 2007. These individuals were then mailed a structured questionnaire, and 1955 (54.6%) returned completed questionnaires and their written informed consent by mail. Among the respondents, 980 (50.1%) were men and 975 (49.9%) were women. Furthermore, 1222 (62.5%) were aged 65 to 74 years and 733 (37.5%) were aged 75 to 84 years; mean age (SD) was 73.8 (5.3) years. In the event of an incomplete answer on a questionnaire, a trained staff person collected the missing information from each study participant by telephone.

Among the 1628 nonresponders, 28 refused to answer the questionnaire, 1591 did not return the informed consent form, 1 person was outside the age range, and 8 questionnaires were completed by someone other than the intended participant because of the physical or mental impairment of the participant.

The questionnaire used for baseline assessment of the 1955 participants comprised 54 simple questions on, among other topics, current and past involvement in hobby activities, self-perceived health status, smoking and drinking habits, self-reported body height and weight, past medical history. The 4 questions on hobby activities were as follows: (1) Do you participate in solitary physical activities (walking, fishing, light gymnastics, etc)? (Yes/no), (2) Do you participate in group physical activities (baseball, ballroom dancing, golf, etc)? (Yes/no), (3) Do you participate in solitary cultural activities (music appreciation, ceramics, handicrafts, etc)? (Yes/no), (4) Do you participate in group cultural activities (cultural club, tea klatch, Japanese chess, etc)? (Yes/no). According to the standard of the Japanese Society for the Study of Obesity, body mass index (BMI, kg/m^2^) was classified into 3 categories—lean, normal, or obese—which correspond to a BMI of less than 18.5, 18.5 to 24.9, or 25.0 or higher, respectively. We conducted a follow-up survey once each year between September 2007 and May 2010 under the terms of a contract between the local governments and our university.

In total, for the assessment of all-cause mortality and incident frailty, 1955 study participants completed questionnaires 3 times during the entire study period (until the end of June 2010). Incident frailty was defined as being newly institutionalized or bedridden at home because of physical disability or severe cognitive impairment. During the follow-up period, 30, 26, and 5 deaths were recorded in September 2008, September 2009, and May 2010, respectively. In addition, 28, 20, and 7 cases of incident frailty were recorded in September 2008, September 2009, and May 2010, respectively. Thirty-seven participants moved away from the study areas, and 1 participant declined continued follow-up. All other participants were followed-up for mortality and frailty until the end of June 2010 and were regarded as alive at the end of observation. Although cause of death was specified by family members (cancer in 19 participants, heart failure in 3, accident in 3, and various other diseases in 4 participants), cause of death could not be ascertained for 32 deaths. Furthermore, although cause of incident frailty was specified by family members (cerebrovascular disease in 11 participants, senile dementia in 8, Parkinson disease in 4, and various other diseases in 19 participants), it could not be determined for 13 participants.

To clarify the effects of each of the 4 categories of hobby activities on death and incident frailty, we divided participants into those who did and did not participate in each type of hobby activity and compared hazard ratios (HRs) by using the Cox proportional hazards model.^19^ Moreover, to assess the effects on death and incident frailty of taking part in 2 or more hobby activities, we divided participants into 3 groups according to the number of hobby activities they reported, ie, 0, 1, or 2 or more hobby activities, and evaluated them separately by their participation in solitary, group, physical, and cultural activities.

The HRs and 95% CIs for death and incident frailty were computed after adjustment for potential confounding factors. The significance level was set at 0.05 or less. Windows SPSS version 15.0J (SPSS, Chicago, IL, USA) was used for all analysis.

## RESULTS

Ten participants developed frailty before the baseline survey in September 2007; thus, data from the remaining 1945 participants were included in the analysis. Table 1 shows the HRs and 95% CIs for all-cause mortality with regard to age, sex, self-perceived health status, smoking and drinking habits, BMI, and past medical history. The risk for all-cause mortality in participants aged 75 years or older was significantly higher than in those younger than 75 years (HR = 2.29; 95% CI 1.38, 3.80). In addition, the risk was significantly lower in women (0.36; 0.20, 0.63) than in men. Moreover, the risk for all-cause mortality in participants with fair (2.30; 1.02, 5.21) or poor (6.66; 2.83, 15.67) self-perceived health was significantly higher than in those with good self-perceived health. The risk for all-cause mortality in participants who drank alcoholic beverages every day (0.47; 0.23, 0.99) was significantly lower than in those who did not, after adjusting for age, sex, and self-perceived health status. Furthermore, the risk for all-cause mortality in participants who had a BMI lower than 18.5 (3.76; 1.87, 7.53) or a past history of cancer (1.96; 1.11, 3.46) was significantly higher than in those who did not, after adjusting for age, sex, and self-perceived health status.

**Table 1.** Hazard ratios (HRs) and 95% CIs for all-cause mortality with regard to age, sex, self-perceived health status, smoking and drinking habits, body mass index (BMI), and past medical history

Variables		No. of deaths	Person-months	Mortality rate^a^	Crude HR	95% CI	Adjusted HR^b^	95% CI
Age, years	65–74	26	36 762	7.1	1.00			
	75–84	35	21 543	16.2	2.29	1.38, 3.80		

Sex	Male	45	29 120	15.5	1.00			
	Female	16	29 185	5.5	0.36	0.20, 0.63		

Self-perceived health status	Good	7	16 578	4.2	1.00			
	Fair	33	34 199	9.6	2.30	1.02, 5.21		
	Poor	21	7466	28.1	6.66	2.83, 15.67		

Smoking habit	No	30	39 713	7.6	1.00		1.00	
	Ex-smoker	21	11 310	18.6	2.47	1.42, 4.32	1.37	0.74, 2.55
	Current smoker	10	7282	13.7	1.81	0.88, 3.70	1.39	0.66, 2.95
					Trend, *P* = 0.120		Trend, *P* = 0.304	
Habit of drinking alcoholic beverages	No	40	32 131	12.4	1.00		1.00	
	Sometimes	11	13 734	8.0	0.65	0.33, 1.26	0.56	0.28, 1.11
	Every day	10	12 411	8.1	0.65	0.33, 1.30	0.47	0.23, 0.99
					Trend, *P* = 0.142		Trend, *P* = 0.026	
BMI	<18.5	11	2515	43.7	5.06	2.57, 9.96	3.76	1.87, 7.53
	18.5–24.9	35	39 909	8.8	1.00		1.00	
	≥25.0	14	15 838	8.8	1.01	0.54, 1.87	1.11	0.60, 2.06

Past medical history								
Hypertension	No	23	29 385	7.8	1.00		1.00	
	Yes	38	28 920	13.1	1.68	0.999, 2.81	1.30	0.77, 2.19
Diabetes mellitus	No	45	48 019	9.4	1.00		1.00	
	Yes	16	10 286	15.6	1.65	0.93, 2.92	1.28	0.72, 2.28
Heart disease	No	44	47 874	9.2	1.00		1.00	
	Yes	16	10 431	15.3	1.66	0.94, 2.94	1.00	0.55, 1.80
Cerebrovascular disease	No	52	54 144	9.6	1.00		1.00	
	Yes	7	4107	17.0	1.76	0.80, 3.88	0.99	0.44, 2.21
Cancer	No	42	50 455	8.3	1.00		1.00	
	Yes	17	7829	21.7	2.62	1.49, 4.61	1.96	1.11, 3.46
Chronic arthritis pain	No	40	37 460	10.7	1.00		1.00	
	Yes	21	20 845	10.1	0.95	0.56, 1.61	0.74	0.43, 1.27

^a^Per 10 000 person-months.

^b^Adjused for sex, age, and self-perceived health status.

Table 2 shows the HRs and 95% CIs for incident frailty with regard to age, sex, self-perceived health status, smoking and drinking habits, BMI, and past medical history. The risk of incident frailty among women was significantly higher than in men (HR = 3.52; 95% CI 2.00, 6.18). The risk for incident frailty among participants with poor self-perceived health was significantly higher than in those with good self-perceived health (7.86; 3.17, 19.46). The risk for incident frailty among participants who occasionally used to drink alcoholic beverages was significantly lower (0.26; 0.08, 0.85) than in those who did not, after adjusting for age, sex, and self-perceived health status. Moreover, the risk for incident frailty among participants who had a BMI less than 18.5 (2.25; 1.03, 4.89) or a past history of cerebrovascular disease (2.45; 1.24, 4.84) was significantly higher than that in those who did not, after adjusting for age, sex, and self-perceived health status.

**Table 2.** Hazard ratios (HRs) and 95% CIs for incident fraily with regard to age, sex, self-perceived health status, smoking and drinking habits, body mass index (BMI), and past medical history

Variables		No. of frail	Person-months	Rate of frailty^a^	Crude HR	95% CI	Adjusted HR^b^	95% CI
Age, years	65–74	23	29 120	7.9	1.00			
	75–84	32	29 185	11.0	1.39	0.82, 2.38		

Sex	Male	18	36 762	4.9	1.00			
	Female	37	21 543	17.2	3.52	2.00, 6.18		

Self-perceived health status	Good	6	16 478	3.6	1.00			
	Fair	28	33 570	8.3	2.27	0.94, 5.47		
	Poor	21	7179	29.3	7.86	3.17, 19.46		

Smoking habit	No	39	39 713	9.8	1.00		1.00	
	Ex-smoker	14	11 310	12.4	1.26	0.68, 2.31	1.28	0.62, 2.63
	Current smoker	2	7282	2.7	0.28	0.07, 1.16	0.36	0.09, 1.54
					Trend, *P* = 0.224		Trend, *P* = 0.368	
Habit of drinking alcoholic	No	39	32 131	12.1	1.00		1.00	
beverages	Sometimes	3	13 734	2.2	0.18	0.06, 0.58	0.26	0.08, 0.85
	Every day	13	12 411	10.5	0.86	0.46, 1.61	1.55	0.72, 3.35
					Trend, *P* = 0.215		Trend, *P* = 0.681	
BMI	<18.5	8	2515	31.8	3.48	1.62, 7.47	2.25	1.03, 4.89
	18.5–24.9	37	39 909	9.3	1.00		1.00	
	≥25.0	9	15 838	5.7	0.61	0.30, 1.27	0.64	0.31, 1.33

Past medical history								
Hypertension	No	21	29 385	7.1	1.00		1.00	
	Yes	34	28 920	11.8	1.65	0.96, 2.85	1.16	0.66, 2.01
Diabetes mellitus	No	41	48 019	8.5	1.00		1.00	
	Yes	14	10 286	13.6	1.60	0.87, 2.94	1.28	0.70, 2.37
Heart disease	No	37	47 874	7.7	1.00		1.00	
	Yes	18	10 431	17.3	2.25	1.28, 3.95	1.35	0.75, 2.43
Cerebrovasuclar disease	No	43	54 144	7.9	1.00		1.00	
	Yes	11	4107	26.8	3.42	1.76, 6.62	2.45	1.24, 4.84
Cancer	No	43	50 455	8.5	1.00		1.00	
	Yes	11	7829	14.1	1.65	0.85, 3.21	1.31	0.67, 2.55
Chronic pain of arthritis	No	31	37 460	8.3	1.00		1.00	
	Yes	24	20 845	11.5	1.39	0.82, 2.38	0.88	0.51, 1.53

^a^Per 10 000 person-months.

^b^Adjused for sex, age, and self-perceived health status.

The Spearman rank correlation coefficient was −0.089 between solitary physical activities and group physical activities, 0.225 between solitary physical activities and solitary cultural activities, −0.009 between solitary physical activities and group cultural activities, −0.031 between group physical activities and solitary cultural activities, 0.271 between group physical activities and group cultural activities, and 0.079 between solitary cultural activities and group cultural activities.

Table 3 shows the HRs and 95% CIs for all-cause mortality with regard to participation in hobby activities, after adjusting for potential confounding factors (age, sex, self-perceived health status, smoking and drinking habits, and past history of cancer) that were significantly associated with the risk of all-cause mortality, as shown in Table 1. None of the risk factors for all-cause mortality associated with participation in solitary physical (HR = 0.70, 95% CI 0.41, 1.18), group physical (1.12; 0.60, 2.09), solitary cultural (0.94; 0.54, 1.65), or group cultural activities (0.73; 0.38, 1.39) significantly decreased after adjustment.

**Table 3.** Hazard ratios (HRs) and 95% CIs for all-cause mortality associated with participation in hobby activities

Variables		No. of deaths	Person-months	Mortality rate^a^	Crude HR	95% CI	Adjusted HR^b^	95% CI
Solitary physical activities	No	24	20 900	11.5	1.00		1.00	
	Yes	37	37 334	9.9	0.86	0.52, 1.44	0.70	0.41, 1.18
Group physical activities	No	48	43 572	11.0	1.00		1.00	
	Yes	13	14 662	8.9	0.81	0.44, 1.49	1.12	0.60, 2.09
Solitary cultural activities	No	42	37 036	11.3	1.00		1.00	
	Yes	19	21 198	9.0	0.79	0.46, 1.36	0.94	0.54, 1.65
Group cultural activities	No	49	38 827	12.6	1.00		1.00	
	Yes	12	19 407	6.2	0.49	0.26, 0.92	0.73	0.38, 1.39

^a^Per 10 000 person-months.

^b^Adjused for sex, age, self-perceived health status, history of cancer, smoking and drinking habits, and BMI.

Table 4 shows HRs and 95% CIs for incident frailty with regard to participation in hobby activities, after adjusting for the potential confounding factors of age, sex, self-perceived health status, smoking and drinking habits, and past history of cerebrovascular disease, since all these potential confounders, except for age, were significantly associated with the risk of all-cause mortality, as shown in Table 2. The risk for incident frailty among respondents who participated in solitary physical activities was significantly lower than in those who did not, even after adjustment (HR = 0.55; 95% CI 0.32, 0.96). Furthermore, the risk for incident frailty among respondents who took part in group cultural activities was significantly lower than in those who did not, even after adjustment (0.40; 0.19, 0.85). Although the risks for incident frailty among respondents who participated in group physical activities (0.58; 0.26, 1.30) and solitary cultural activities (0.74; 0.40, 1.35) were lower, the HRs were not statistically significant after adjustment. Moreover, in additional analysis that excluded events that were observed less than 6 months after baseline, so as to exclude a potential effect at the baseline, the effect of group cultural activity remained statistically significant (0.45; 0.21, 0.97).

**Table 4.** Hazard ratios (HRs) and 95% CIs for incident frailty associated with participation in hobby activities

Variables		No. of cases of incident frailty	Person-months	Rate of frailty^a^	Crude HR	95% CI	Adjusted HR^b^	95% CI
Solitary physical activities	No	25	20 900	12.0	1.00		1.00	
	Yes	29	37 334	7.8	0.65	0.38, 1.11	0.55	0.32, 0.96
Group physical activities	No	47	43 572	10.8	1.00		1.00	
	Yes	7	14 662	4.8	0.44	0.20, 0.98	0.58	0.26, 1.30
Solitary cultural activities	No	39	37 036	10.5	1.00		1.00	
	Yes	15	21 198	7.1	0.67	0.37, 1.22	0.74	0.40, 1.35
Group cultural activities	No	46	38 827	11.8	1.00		1.00	
	Yes	8	19 407	4.1	0.35	0.16, 0.74	0.40	0.19, 0.85

^a^Per 10 000 person-months.

^b^Adjused for sex, age, self-perceived health status, cerebrovascular disease, smoking and drinking habits, and body mass index.

Table 5 shows the relationship between number of hobby activities and incident frailty for each category of activity. Regarding solitary activities, the HRs for incident frailty among those participating in 1 solitary hobby activity, as well as those participating in 2 solitary activities, showed a significant decrease with the number of solitary activities (trend *P* = 0.048). Similarly, regarding group physical and cultural hobby activities, the HRs for incident frailty among those participating in 1 and 2 hobby activities also showed a significant decrease in association with the number of hobby activities (trend *P* = 0.017, *P* = 0.012, and *P* = 0.021, respectively).

**Table 5.** Hazard ratios (HRs) and 95% CIs for incident frailty associated with number of hobby activities

Variables	No. of activities	No. of cases of incident frailty	Person-months	Rate of frailty^a^	Crude HR	95% CI	Adjusted HR^b^	95% CI
Solitary activities (physical or cultural)	0	21	16 312	12.9	1.00		1.00	
	1	22	25 312	8.7	0.68	0.37, 1.23	0.59	0.32, 1.08
	2	11	16 610	6.6	0.51	0.25, 1.07	0.49	0.24, 1.03
					Trend *P* = 0.064		Trend *P* = 0.048	

Group activities (physical or cultural)	0	41	32 258	12.7	1.00		1.00	
	1	11	17 883	6.2	0.48	0.25, 0.94	0.57	0.29, 1.11
	2	2	8093	2.5	0.19	0.05, 0.80	0.26	0.06, 1.10
					Trend *P* = 0.003		Trend *P* = 0.017	

Physical activities (solitary or group)	0	24	14 463	16.6	1.00		1.00	
	1	24	35 546	6.8	0.41	0.23, 0.71	0.40	0.22, 0.72
	2	6	8225	7.3	0.44	0.18, 1.07	0.47	0.19, 1.18
					Trend *P* = 0.007		Trend *P* = 0.012	

Cultural activities (solitary or group)	0	34	25 756	13.2	1.00		1.00	
	1	17	24 351	7.0	0.53	0.29, 0.94	0.60	0.33, 1.09
	2	3	8127	3.7	0.28	0.09, 0.91	0.33	0.10, 1.09
					Trend *P* = 0.005		Trend *P* = 0.021	

^a^Per 10 000 person-months.

^b^Adjused for sex, age, self-perceived health status, history of cerebrovascular disease, smoking and drinking habits, and body mass index.

## DISCUSSION

In this follow-up survey from September 2007 to May 2010, we assessed the relationships of hobby activities with all-cause mortality and incident frailty among elderly adults. We found that participation in group cultural activities was associated with a significantly lower risk of incident frailty, after adjusting for potential confounders. Moreover, in additional analysis that excluded events observed less than 6 months after baseline, so as to exclude a potential effect at the baseline,^20^ the effects of group cultural activity remained statistically significant.

Several mechanisms might explain why participation in group cultural activities has health benefits. First, participating in activities with others might reduce feelings of loneliness and increase mental and physical fitness, which could decrease the risk of frailty. It could also be that individuals in group activities receive encouragement from others and are more likely to continue participation as compared with solitary activities. Jung et al^21^ reported that participation in social activities was significantly associated with a lower risk of incident frailty among elderly people. Iwasaki et al^22^^,^^23^ found that men who did not participate in community groups and had no close friendships with their neighbors had a significantly higher risk of death. Jylhä and Aro^24^ reported that social participation in activities such as family ceremonies and religious events was associated with a significantly lower risk of all-cause mortality among elderly people. Gognalons-Nicolet et al^25^ reported that elderly people who did not participate in clubs or associations had a significantly higher mortality rate. In addition, House et al^26^ found that men reporting a higher level of social relationships and activities were significantly less likely to die.

Second, social environments that allow easy participation in group activities might give elderly adults opportunities to maintain good health. Hyyppä et al^27^ noted that higher social capital, such as voluntary associational activities and friendship networks, was significantly associated with good self-rated health. In addition, Hanson et al^28^ observed a higher mortality rate among elderly men with low availability of emotional support.

Third, participation in cultural activities may itself have a direct effect on good health. Wilkinson et al^18^ observed a significant association between participation in cultural activities and good self-reported health. Wilson et al^17^ indicated that frequent participation in cognitively stimulating activities, eg, playing games, was associated with a significantly lower risk of Alzheimer disease. Bygren et al^14^ showed that frequent attendance at cultural events significantly reduced mortality among individuals aged 16 to 74 years. Konlaan et al^15^ observed significantly higher mortality among people aged 25 to 74 years who rarely visited cinemas, concerts, museums, or art exhibitions, as compared with those who frequently did so. Väänänen et al^16^ found that high engagement in cultural activities was associated with significantly lower all-cause mortality among full-time industrial employees aged 18 to 65 years, although we observed no association between cultural activities and all-cause mortality in the present study.

We also found that solitary physical activities were associated with a significantly reduced risk of incident frailty after adjusting for potential confounders. Because solitary participation in physical activities is probably easier than group participation in such activities, a larger proportion of our study participants participated in solitary physical activities, as shown in Tables 3 and 4. Similarly, Lennartsson and Silverstein^7^ reported that participation in solitary-active activities was significantly associated with reduced mortality risk among men. Everard et al^12^ reported that leisure activities were significantly associated with higher health scores. Bucksch^9^ found that, as compared with a sedentary lifestyle, moderately intense lifestyle activity had a significant protective dose–response relationship with relative risk.

A number of reports have shown a significant effect of physical activity on good health status regardless of whether the activity was performed alone or in a group. Okano et al^13^ showed that maintaining regular moderate or vigorous physical activity was significantly associated with good self-perceived health and fitness among Japanese male employees. Paffenbarger et al^5^ reported that starting moderately vigorous sports activities was significantly associated with a reduced risk of mortality. Leitzmann et al^10^ found that moderate or vigorous exercise was significantly associated with a reduced risk of mortality. Stessman et al^11^ observed that participation in vigorous physically activity was significantly associated with lower mortality. Lee et al^8^ also found that men who regularly participated in physical activity had a significantly lower mortality rate. In addition, Lee et al^6^ reported that total energy expenditure during vigorous activity was significantly inversely related to mortality among middle-aged men.

In this study, there was no statistically significant association of group physical activity or solitary cultural activity with all-cause mortality or incident frailty, but, except for the HR for all cause mortality associated with group physical activities, the HRs of these hobby activities did reflect a tendency toward risk reduction for both activity types, as in other activities.

There was an increase in the HR for all-cause mortality risk associated with group physical activities. Our explanation for this finding is as follows. Because of the small number of events, we divided participants into 2 groups—those who did and did not participate in each type of hobby activity—and estimated the HR for individuals who participated in the activity. Therefore, the reference group included individuals who took part in no activities as well as those who participated in other hobby activities. Group physical activity was the least frequent hobby activity in this study, and the reference group included larger numbers of individuals with other hobby activities.

The associations of group physical and solitary cultural activities with both endpoints might have been statistically significant for a larger cohort or a longer follow-up period. Therefore, a large-scale cohort study is necessary to confirm the effects of group physical and solitary cultural activities.

Our analysis of the number of hobby activities showed that each increase in the number of such activities significantly decreased the risk for incident frailty. Therefore, cultural and solitary activities could potentially reduce the risks of incident frailty as much as physical and group activities among elderly adults.

There were some limitations in our study. Although the response rate for the follow-up survey of the study participants was satisfactorily high, that of the baseline survey was around 55%. Thus, the possibility of selection bias should be considered. The 7 study areas were chosen, not by random sampling, but because they were readily accessible from our university. Therefore, the generalizability of our results is uncertain.

Under the terms of the contract between the local governments and our university, the duration of follow-up for this cohort was limited to 3 years. The fact that none of the 4 categorized activities was found to be a preventive factor for all-cause mortality might have been due to the limited sample size of study participants and/or the limited follow-up period. Therefore, a larger population study or an extended follow-up survey is needed to confirm the present results. In addition, we did not determine whether incident frailty preceded death among participants who died; thus, we might have overlooked cases of incident frailty. Furthermore, because we did not conduct a follow-up survey of participants who become frail, we might have overlooked subsequent deaths among those individuals.

We used self-reported weight and height, which are subject to error. However, self-reported weight and height were found to be highly valid as compared with measured values.^29^ In this study, family members reported incident frailty on behalf of study participants when the participant was newly institutionalized or bedridden at home due to physical disability or severe cognitive impairment. Therefore, cases of incident frailty might include instances in which a participant was institutionalized for his/her family’s social and economic reasons and not necessarily due to a decline in activities of daily living.

Participation in group cultural activities and solitary physical activities was significantly associated with decreased incident frailty. However, these findings must be validated in future studies.
